# Variance, Genetic Control, and Spatial Phenotypic Plasticity of Morphological and Phenological Traits in *Prunus spinosa* and Its Large Fruited Forms (*P. x fruticans*)

**DOI:** 10.3389/fpls.2016.01641

**Published:** 2016-11-03

**Authors:** Kristine Vander Mijnsbrugge, Arion Turcsán, Leander Depypere, Marijke Steenackers

**Affiliations:** ^1^Department of Forest Genetic Resources, Research Institute for Nature and ForestGeraardsbergen, Belgium; ^2^Department of Biometrics and Agricultural Informatics, Szent István UniversityBudapest, Hungary; ^3^Department of Forest Reproductive Material and Plantation Management, Institute of Silviculture and Forest Protection, West-Hungarian UniversitySopron, Hungary; ^4^Formerly affiliated with the Department of Biology, Research Group Spermatophytes, Ghent UniversityGhent, Belgium

**Keywords:** crop-to-wild gene flow, blackthorn, sloe, damson plum, bud burst, flower opening, leaf senescence, general linear mixed models

## Abstract

*Prunus spinosa* is a highly esteemed shrub in forest and landscape plantings. Shrubs with larger organs occur often and are considered either as large fruited forms of *P. spinosa* or as *P. x fruticans*, involving a hybridization process with the ancient cultivated *P. insititia* (crop-to-wild gene flow). As climate change may augment hybridization processes in the future, a hybrid origin is important to detect. In addition, studying crop-to-wild gene flow can give insights in putative consequences for the wild populations. We studied the *P. spinosa*–*P. x fruticans* group, focusing on morphology and phenology in three experimental plantations. Two plantings harbored cuttings of *P. spinosa* (clone plantations). A third plantation comprised of a half-sib offspring from a population with both *P. spinosa* and *P. x fruticans* (family plantation). Several results point to a hybridization process as the origin of *P. x fruticans*. The clone plantation revealed endocarp traits to be more genetically controlled than fruit size, while this was the opposite in the family plantation, suggesting the control of fruit size being derived from the putative *P. insititia* parent. Bud burst, flower opening, and leaf fall were genetically controlled in the clone plantation, whereas in the family plantation intrafamily variability was remarkably large for the bud burst and leaf fall, but not for the flower opening. This suggests there is a reduced genetic control for the first two phenophases, possibly caused by historic hybridization events. Pubescence on the long shoot leaves in the family plantation deviated from the short shoot leaves on the same plants and from long and short shoot leaves in the clone plantation, suggesting again a *P. insititia* origin. Finally, we quantified spatial phenotypic plasticity, indicating how *P. spinosa* may react in a changing environment. In contrast to the bud burst and leaf fall, flower opening did not demonstrate plasticity. The fruit size was diminished at the growth site with the shortest growing season while interestingly, the leaf width was enlarged. Leaf size traits appeared more plastic on the long shoots compared to the short shoots, although partitioning of variance did not display a lesser genetic control.

## Introduction

Species of the subgenus *Prunus* within the stone fruit genus *Prunus* L. have for long attracted interest of many systematists, botanists, and geneticists as it comprises of wild species with a considerable ecological importance, providing plenty of ecosystem services, as well as economically valuable fruit trees. In this subgenus of European and Asian plums (section *Prunus*), North American plums (section *Prunocerasus*), and apricots (section *Armeniaca*), most species are diploids, whereas some are tetraploids or hexaploids. Polyploidization, hybridization and introgression, and centuries of domestication and cultivation have contributed to the complexity of this polymorphic group of species (Stebbins, 1950). Phylogenetic analyses revealed a clear differentiation between the section *Prunus* and other sections within the subgenus *Prunus* (Shaw and Small, 2004; Bortiri et al., 2006), and the presence of four clades within this section (Reales et al., 2010), of which one clade is comprised of the related wild species *P. spinosa* and the domesticated species *P. insititia* as well as the *P. domestica*. Still, the relationship between these taxa is not yet fully understood. *P. spinosa* (black thorn) is an allotetraploid. It is widely distributed in European deciduous forests and in open farmland (Woldring, 2000). It is pollinated by insects, dispersed by birds (Guitian et al., 1993; Körber-Grohne, 1996) and is able to propagate vegetatively through root suckers (Leinemann et al., 2014). *P. spinosa* is known as a morphologically variable species and this variability is likely strengthened by hybridization with escapes from cultivated forms (Hanelt, 1997). Amplified fragment length polymorphism (AFLP) analyses, a neutral molecular marker that is biparentally inherited, has shown a relatively large differentiation between the natural populations compared to other woody species (Vander Mijnsbrugge et al., 2013; Leinemann et al., 2014). It is suggested that founder effects (long-distance seed dispersal through birds) together with vegetative propagation may cause this relatively high interpopulation heterogeneity (Leinemann et al., 2014). In the long-lived clonal species with reduced sexual recruitment, particular genotypes may turn out to be more successful than others and can displace those that are less competitive (Leinemann et al., 2014). In addition, it has been speculated that the long-distance seed dispersal in *P. spinosa* through birds has contributed to the low interpopulation diversity observed with chloroplast DNA analyses (maternally inherited neutral marker), erasing phylogeographic genetic structures (Mohanty et al., 2002; Leinemann et al., 2014). Planted *P. spinosa* populations with stock of non-local material may have also contributed to the relatively high interpopulation differentiation based on the AFLP markers as to the relatively low interpopulation differentiation based on cpDNA markers (Leinemann et al., 2014).

*Prunus insititia* L. (damson plum) is a cultivated plum which is regarded as a subspecies of *P. domestica* (Bailey, 1925; Browicz, 1972) or as the same taxon (Woldring, 2000). *P. domestica* L., which is one of the most widely cultivated plums, has never been found in the wild and its origin is subject to a long standing debate. Both *P. insititia* and *P. domestica* are since long considered to have originated from an allopolyploidization (interspecific hybridization followed by polyploidization) between a diploid *P. cerasifera* Ehrh. and a tetraploid *P. spinosa* (Stebbins, 1950). An evolvement from a hexaploid form of *P. cerasifera* has also been hypothesized (Zohary, 1992). *P. insititia* is not floristically indigenous to Belgium, but the endocarps are widely available in archeological finds from the late middle ages onward (will be published elsewhere). *P. insititia* is thought to have originated in western Asia or southern Russia and subsequently to have spread over Europe and larger parts of Asia by cultivation, possibly already in Neolithic times (Körber-Grohne, 1996; Woldring, 2000). Hybridization between *P. spinosa* (narrow leaves, small and round fruits and endocarps, shrub) and *P. insititia* (wide leaves, large and flattened fruits and endocarps, small tree) is suggested to occur, resulting in the taxon *P. x fruticans* Weihe (Webb, 1968; Körber-Grohne, 1996; Woldring, 2000; Maes, 2013). According to Hanelt (1997), *P. x fruticans* is difficult to distinguish from true *P. spinosa*, and may be an old abandoned fruit crop. Still, some taxonomists doubt the hybrid origin and consider these shrubs to be intermediate or deviating characters as a morphological variety of *P. spinosa* (Werneck and Bertsch, 1959; Fournier, 1977). A neutral molecular marker analysis (AFLP) did not differentiate *P. spinosa* from *P. x fruticans*, while a clear distinction was displayed between this group and *P. insititia*, suggesting the variety hypothesis (Depypere et al., 2009). Large and wide leaved shrubs of *P. spinosa* in a provenance trial tended to flush and flower slightly earlier and to grow slightly taller compared to smaller and narrower leaved *P. spinosa*, and this minor differentiation was hypothesized to be a legacy of historical gene flow (hybridization followed by back crossings) of domesticated *P. insititia* in natural *P. spinosa* populations (Vander Mijnsbrugge et al., 2016). As *P. insititia* has been domesticated since long as a fruit tree, this putative hybridization may be considered as a historical crop-to-wild gene flow and may have occurred already for many generations and possibly on many occasions, with no evident indications to negative consequences for the fitness of the natural *P. spinosa* populations (Vander Mijnsbrugge et al., 2016).

As *P. spinosa* is a wild and indigenous species found in many European countries, for which conservation measures are taken (Vander Mijnsbrugge et al., 2005; Kleinschmit et al., 2008; Kjær et al., 2009), its large fruited form displaying traces of crop-to-wild gene flow from the cultivated *P. insititia*, may complicate the principle of conservation of a “wild” species. Nowadays, *P. spinosa* is planted in large quantities both in forests as well as in landscapes in many European countries to improve species diversity, to restore historical landscapes and to preserve wildlife habitat. As the European directive regulating marketing of forest trees (EC, 2000) is not obligatory for shrub species, the origin of the seeds used by private nurseries in Western European countries to grow planting stock are often derived from southern and eastern European countries where seed collection is cheaper. Many examples exist in Flanders (northern part of Belgium) of planting stock being a mix of *P. spinosa* and its large fruited forms, in rare occasions even mixed with *P. insititia* types, implying that plantations working toward nature conservation unintentionally may harbor domestic-like types. Our central aim was to enhance our comprehension of the *P. spinosa*–*P. x fruticans* group as previous results were not conclusive whether the large fruited forms of *P. spinosa* are either a variety of *P. spinosa* or originated from a hybridization with *P. insititia* (Depypere et al., 2009; Vander Mijnsbrugge et al., 2016). In addition, we wanted to detect how this species group may react upon a changing environment. We followed two objectives. Firstly, we looked for traces of crop-to-wild gene flow and secondly, we studied plastic responses to different growth environments. To approach related taxa, it is evident that variation of many diagnostic traits has to be studied simultaneously, not relying on single traits (Stebbins, 1950). Apart from the well-studied morphological endocarp, fruit, and leaf traits, we quantified variability also in phenological traits in *P. spinosa* and its large fruited forms. We studied plantations of both clonally replicated material and seedlings.

## Materials and Methods

### Source Material

**FIGURE 1:**
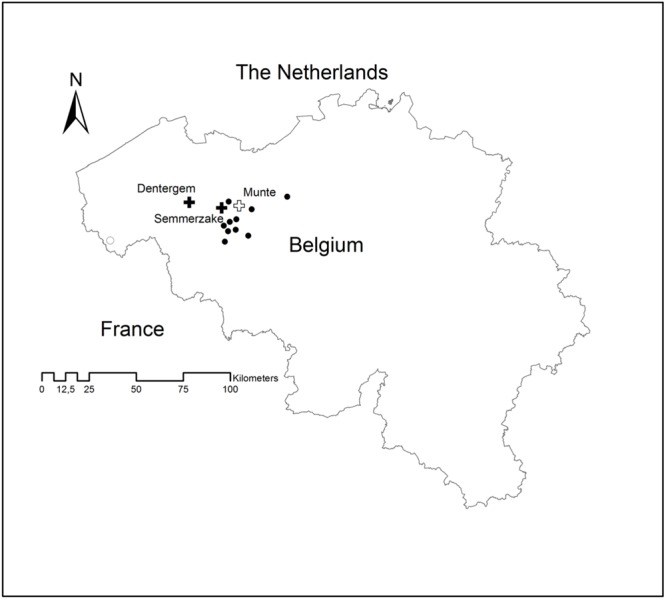
**Map showing the locations of the source populations and the three plantations.** Filled circles: source populations for the clone plantations; open circle: source population for the family plantation; filled cross: clone plantations (Dentergem and Semmerzake); open cross: family plantation (Munte).

For the clonal experiment, cuttings were taken in 2003 from 28 genotypes sampled in 10 populations (**Figure 1**; **Table 1**) that were considered autochthonous in an inventory specifically aiming to locate growth sites with autochthonous populations of woody species in the northern part of Belgium (Vander Mijnsbrugge et al., 2005). In this inventory, small fruited *P. spinosa* are identified as separate from the larger fruited *P. x fruticans* according to Maes (2013). As *P. spinosa* is known to propagate vegetatively by root suckers, care was taken not to sample from the same genotype by evaluating the habitus of the shrubs together with leaf morphology in the field. Rooted cuttings were grown to 126 two-year-old planting stock in the nursery of the Research Institute for Nature and Forest in Geraardsbergen, Belgium, following standard nursery methods. From these, 50 plants were planted in Dentergem (from 26 genotypes, on average 2 ramets per genotype) and 76 in Semmerzake (from 27 genotypes, on average 3 ramets per genotype) with 25 genotypes being represented in both plantations (**Table 1**; **Figure 1**). The location in Semmerzake is characterized by a nutrient rich alluvial soil (altitude of 7.5 m). The shrubs were planted next to a forest (shrubs were south west oriented). In comparison, the soil in Dentergem is less nutrient rich (texture of loamy sand to sand, normal soil moisture, altitude of 24 m), and the shrubs were more exposed as they were planted in between agricultural fields with no forest, wooded bank, hedge, or tree row in the neighborhood as protection against harsh weather conditions. For the half-sib offspring experiment (family experiment), berries were collected per shrub from eight shrubs in 2005 growing in an old farmers hedge in Dranouter, including both small fruited and large fruited *P. spinosa* (**Figure 1**). Seeds were cleaned, stratified, and grown to 2-year-old planting stock following standard nursery methods at the nursery of the Research Institute for Nature and Forest in Geraardsbergen, Belgium. Finally, 46 plants were planted in Munte (**Figure 1**), with an average of six pedigrees per mother shrub. Here, the soil was characterized by a loamy sand texture and a normal soil moisture (altitude of 22 m). Shrubs were planted at a 20 m distance of a forest edge, with the shrubs being south oriented (less exposed conditions). In all three plantations the shrubs were planted at a spacing of 3 m × 3 m and individually mingled (single tree plot design).

**Table 1:** Source material for the clone plantations.

		Dentergem	Semmerzake
Location of source population	*n*_g_	*n*_p_ (*n*_g_)	*n*_p_ (*n*_g_)
Brakel	2	2 (1)	4 (2)
Ename	5	9 (4)	15 (5)
Horebeke	1	2 (1)	3 (1)
Lebbeke	1	2 (1)	0 (0)
Lierde	4	11 (4)	15 (4)
Mater	3	6 (3)	9 (3)
Merelbeke	4	8 (4)	14 (4)
Schorisse	5	6 (5)	11 (5)
Sint-Lievens-Houtem	1	1 (1)	2 (1)
Zwalm	2	3 (2)	3 (2)

*Indicated are the location of the source population, the number of genotypes that were vegetatively propagated through cuttings (*n*_g_), and the number of plants planted in each clone plantation (*n*_p_).*

### Sampling, Measuring, and Scoring Morphological and Phenological Traits

In the three plantations several morphological traits were measured and phenological variables were scored in 2015. At this time, plants had grown to well-developed shrubs and the vast majority was flowering and fructifying. For the morphological variables (**Table 2**) two leaves were carefully selected, one from a short shoot, which was located at breast height (between 1.3 and 1.5 m) and in the shadow of its own or neighboring shrubs, and one leaf from a long shoot, for which a high reaching branch at the top of the shrub was sampled which was fully exposed to the sun. For both shoots, a fully developed and damage free leaf was taken for herbarium which was the fourth to sixth leaf counting from the top of the shoot. For each leaf, the length and the widest width of the lamina was measured with a caliper. At the lower side of the leaves, pubescence was scored on the central vein following a 6-level scale going from glabrous (score 1) to densely pubescent (score 6). From each shrub two fully developed and damage free berries were sampled on a fully exposed and fructifying branch at the top of the shrub. From each berry the widest width was measured with a caliper. For (slightly) elongated berries, the widest width was simply the length of a berry. As round berries can have a greater width than length, the largest measure was looked for in any direction and called widest width. After this, the mesocarp (flesh of fruit) was removed and the endocarp (stone) was cleaned with water and air dried. Then stone length, width and thickness were measured with a caliper. An average was taken from the two measurements per shrub for fruit and for stone traits.

**Table 2:** Description of morphological leaf and fruit traits.

Trait	Description
SL	Length of endocarp (mm)
SW	Width of endocarp (mm)
ST	Thickness of endocarp (mm)
FWW	Widest width of berry (mm)
LLs	Lamina length, on short shoot (mm)
LWs	Lamina width, on short shoot (mm)
LPs	Pubescence on central vein, lower side of leaf on short shoot
LLl	Lamina length, on long shoot (mm)
LWl	Lamina width, on long shoot (mm)
LPl	Pubescence on central vein, lower side of leaf on long shoot

Bud burst, flower opening and leaf shedding were scored following a 6-level, 7-level, and 5-level protocol, respectively (**Table 3**). For all phenophases, the whole shrub was evaluated and a mean score level was given. Bud burst and flower opening were scored in the clone plantation in Dentergem on April 1 and April 20 whereas leaf fall was scored on September 29 and October 13. In the clone plantation in Semmerzake, bud burst and flower opening were scored on March 26, April 9, and April 16, while leaf fall was scored on September 29 and October 13. Days of observation in the family plantation in Munte for bud burst and flower opening were March 26, April 9, and April 16, and for leaf fall September 23 and October 14. All basic data are presented in Supplementary Tables S1 and S2.

**Table 3:** Description of bud burst, flower opening, and leaf shedding score levels.

Phenophase	Score level	Description
Bud burst (BB)	1	Buds in rest
	2	Swollen buds
	3	First leaves start to protrude but not yet unfolding
	4	First leaves unfolding (up to 25%)
	5	25–75 of the leaves unfolding
	6	More than 75% of the leaves unfolding
Flower opening (FO)	1	Buds in rest
	2	Swollen buds still green
	3	Swollen buds turning to white
	4	First flowers opening (less than 25%)
	5	Between 25 and 75% of flowers opening
	6	Between 75 and 100% of flowers opening
	7	First flowers showing signs of withering
Leaf fall (LF)	1	No leaf shedding
	2	Up to 25% leaves shed
	3	Between 25 and 75% leaves shed
	4	Between 75 and 95% leaves shed
	5	All leaves shed

### Statistical Analysis

All statistical analyses were performed in the open source software R 3.1.2 (R Core Team, 2014). Linear mixed models were applied for the individual morphological traits measured in the clone plantations in the package lme4 (Bates et al., 2015), with each individual morphological trait (T_m_) as response variable, site (S) in the fixed part of the model and genotype (G) in the random part (random intercept):

(1) $$\begin{array}{c} T_{m}\;=\;\alpha \;+\;\beta_{S\cdot }S(\mathrm{fixed})\;+\;{\mathrm{ranef}}_{G}(\mathrm{random}) \end{array}$$

with α as the intercept of the model, β_S_ as the estimated coefficient for the fixed covariate S and ranef_G_ as the random effect coefficients for all levels of the variable G. Variance components attributable to the variation between the genotypes, further called “intergenotype” (variance of genotype σ^2^_G_) and to the variation within each genotype, thus variance between ramets of a genotype, further called “intragenotype” (variance of the residual error σ^2^_e_) were extracted from the models.

Similarly, linear mixed models were run for the variance components analysis of the morphological traits in the family experiment, with each individual morphological trait (T_m_) as response variable, but without site in the fixed part as the family experiment is planted in only one place. Genotype of the mother shrub from which offspring is derived from, called family (F), resided in the random part (random intercept):

(2) $$\begin{array}{c} T_{m}\;=\;\alpha \;+\;{\mathrm{ranef}}_{F}(\mathrm{random}) \end{array}$$

Variance components attributable to the variation between the offspring of different mother genotypes, further called “interfamily” variance (variance of family σ^2^_F_), and to the variation between the offspring within each mother genotype, further called “intrafamily” (variance of the residual error σ^2^_e_), were extracted from the model.

The phenological traits (T_ph_) were modeled using cumulative logistic regression in the package ordinal (Christensen, 2013). The command clmm in the package ordinal models the chance (p) to maximally have reached a given level of the ordinal response variable. We ordered the score levels of bud burst and flower opening in decreasing order, so that the chance to maximally have reached, e.g., bud burst score 3 included scores 6, 5, 4, and 3. This can be interpreted as having reached at least score 3. The score levels for leaf fall were ordered in increasing order. For the clonal plantations, models were run with each individual phenological trait (T_ph_) as response variable, and site (S) in the fixed part of the model. Here, day (D) was added in the fixed part to account for the different observation days. In the random part (random intercept), genotype (G) was present together with a unique shrub identity code (ID) to account for the repeated measurements on the same plants.

(3) $$\begin{array}{c} \log[p_{\mathrm{Tph}}/(1\;-\;p_{\mathrm{Tph}})]\;=\;\alpha_{T}\;-\;\beta_{D\cdot }D(\mathrm{fixed})\;-\;\beta_{S\cdot }S(\mathrm{fixed})\;-\;{\mathrm{ranef}}_{G}(\mathrm{random})\;-\;{\mathrm{ranef}}_{\mathrm{ID}}(\mathrm{random}) \end{array}$$

with α_T_ as a threshold value indicating the passing on from one level of the ordinal phenological response variable to the next; β_D_ and β_S_ as the estimated coefficients for the fixed covariates D and S; and ranef_G_ and ranef_ID_ as the random effect coefficients for all levels of the variables G and ID. The intergenotype variance component (σ^2^_G_) and the intragenotype variance component (σ^2^_ID_) were extracted from the models. For a correlation analysis among and between the phenological and morphological traits, a day was calculated for each shrub and for each phenological trait based on the presented models applying a threshold value α_T_ for the passing on from level 4 to level 3 (bud burst and flower opening) or from level 2 to level 3 (leaf fall) and a value for p_Tph_ of 50%. In this way, the days were calculated for which half of the plants reached at least a phenological score 3 for bud burst and flower opening, or maximally score 3 for leaf fall.

For the family plantation similar models were run but without the location covariate as it concerned only one plantation:

(4) $$\begin{array}{c} \log[p_{\mathrm{Tph}}/(1\;-\;p_{\mathrm{Tph}})]\;=\;\alpha_{T}\;-\;\beta_{D\cdot }D(\mathrm{fixed})\;-\;{\mathrm{ranef}}_{F}(\mathrm{random})\;-\;{\mathrm{ranef}}_{\mathrm{ID}}(\mathrm{random}) \end{array}$$

The interfamily variance component (σ^2^_F_) and the intrafamily variance component (σ^2^_ID_) were extracted from the model. For the correlation analysis a day was calculated for each shrub, and for each phenological trait based on the presented model in the same way as the clone plantations.

Two principal component analyses were performed on the morphological traits in the two types of plantations: the clones and the families. The three first principal components were subsequently added as covariates in the fixed part of the above-mentioned phenological models to analyze the relationship between morphological traits and the phenological responses.

## Results

### Morphological Traits

**FIGURE 2:**
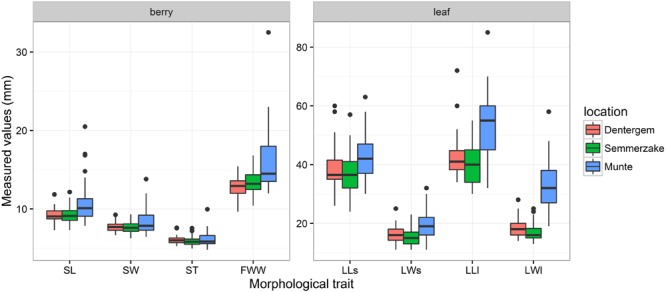
**Boxplots of measured morphological traits in the three plantations.** Dentergem and Semmerzake comprise clonal plants from *P. spinosa* whereas Munte comprises families (half sib seedlings) from *P. spinosa*–*P. x fruticans*. Abbreviations of the traits are in **Table 2**.

**FIGURE 3:**
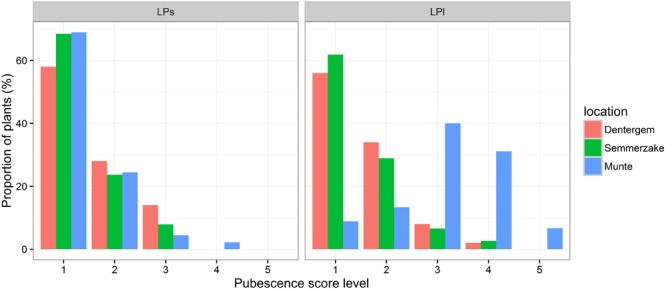
**Proportion of shrubs in the different leaf pubescence score levels in the three plantations.** Dentergem and Semmerzake comprise clonal plants from *P. spinosa* whereas Munte comprises families (half sib seedlings) from *P. spinosa*–*P. x fruticans*. Trait abbreviations are in **Table 2**.

**FIGURE 4:**
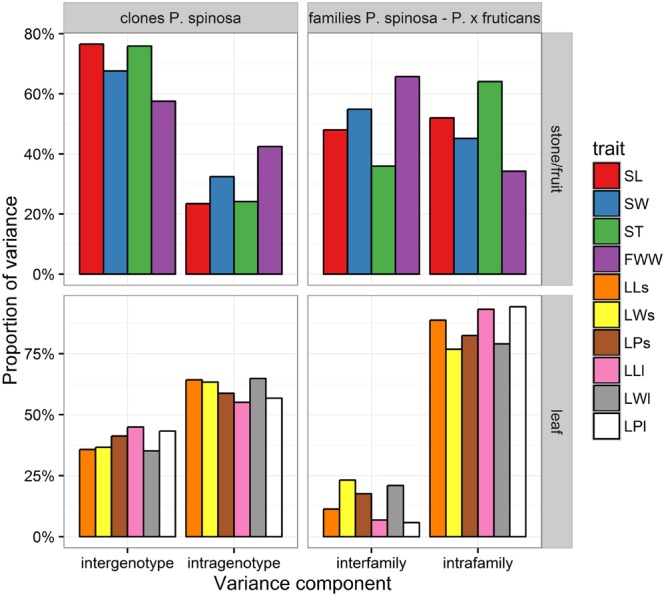
**Partitioning of variance for the individual morphological traits in the clone and family plantations.** Abbreviations of traits are in **Table 2**.

Morphological traits were summarized in boxplots (**Figure 2**) and histograms (**Figure 3**). Evidently, the traits measured in the Munte plantation harboring the families (half-sib offspring) derived from a mixture of *P. spinosa* and *P. x fruticans* shrubs showed all measured endocarp, fruit, and leaf traits as having higher mean values and higher variability, compared to the traits measured in the clone plantations that contain only *P. spinosa*. When considering the endocarp measurements (**Figure 2**), length augmented from *P. spinosa* in the clonal plantations to *P. spinosa*–*P. x fruticans* in the family plantation, the ratio was higher for length (mean ratio SL_families_/SL_clones_ = 1.17) compared to width (mean ratio SW_families_/SW_clones_ = 1.09) and to thickness (mean ratio ST_families_/ST_clones_ = 1.03), implying smaller endocarps being more circular and larger endocarps being more flattened and elongated. Leaves on long shoots in the family plantation tended to have more circular leaf blade shapes (mean ratio LLl/LWl_families_ = 1.71) compared to the short shoot leaves on the same shrubs (mean ratio LLs/LWs_families_ = 2.26) and compared to the leaves from *P. spinosa* shrubs in the clone plantation (mean ratio LL/LW_clones_ = 2.37 for short shoot and 2.39 for long shoot leaves). The pubescence on the central vein at the lower side of the leaves deviated remarkably between on the one hand exposed long shoots in the family plantation and on the other hand the short shoots in the family plantation and the long and short shoots in the clone plantation (**Figure 3**). As ramets were planted in two sites, trait variation between the sites could be attributed to a plastic reaction on the different growth environments in the clone plantations. To quantify this spatial phenotypic plasticity, significance of site as covariate in the fixed part of the mixed models of the individual traits was evaluated (**Table 4**). Fruit widest width expressed the largest spatial plasticity. For the endocarp traits, only thickness displayed significant plasticity between the two sites whereas for the leaf traits the width and length of fully exposed leaves on long shoots were more plastic than leaves on short shoots, with leaf width being more plastic than leaf length. In addition, we partitioned the variance, as quantified by the mixed models, into different components for both the clone and the family plantations. For the clone plantations the variance could be partitioned into intergenotype and intragenotype variations (while the plantation sites were accounted for as covariate in the fixed part of the mixed models): the intragenotype variation was plastic in nature as the ramets have the same genotype, leaving intergenotype variation being genetically determined. In the plantation of the families, interfamily variation, relative to intrafamily variation, gave an indication of genetic control of the traits. Clearly, the endocarp traits displayed the strongest genetic control, followed by the fruit size and finally by the leaf traits, with no clear difference between short shoot and long shoot leaves (**Figure 4**). Similar to the clone plantations, the endocarp traits in the family plantation displayed a stronger genetic control compared to the leaf traits with no difference between short and long shoot leaves. But remarkably dissimilar was fruit size in the family plantation showing a stronger genetic control than the endocarp traits (**Figure 4**).

**Table 4:** Model statistics for the covariate site (clone plantations) in the mixed models with the individual morphological traits as response variables.

Response variable	Covariate	Estimate	Standard error	df	*t*-value	*P* value
SL	Site	0.11	0.10	95	1.12	0.266
SW	Site	-0.12	0.07	95	-1.61	0.110
ST	Site	-0.14	0.05	95	-2.82	**0.0059^∗∗^**
FWW	Site	0.63	0.17	95	3.72	**<0.001^∗∗∗^**
LLs	Site	-0.93	0.99	97	-0.94	0.350
LWs	Site	-0.83	0.40	97	-2.07	**0.0416^∗^**
LPs	Site	-0.18	0.10	97	-1.81	0.074
LLl	Site	-2.09	0.99	97	-2.11	**0.0378^∗^**
LWl	Site	-1.41	0.44	97	-3.20	**0.0019^∗∗^**
LPl	Site	-0.06	0.11	97	-0.55	0.585

*Abbreviations of traits are in **Table 2**. Significant results are in bold: ^∗∗∗^*P* < 0.001; ^∗∗^*P* < 0.01; ^∗^*P* < 0.05.*

### Phenological Traits

**FIGURE 5:**
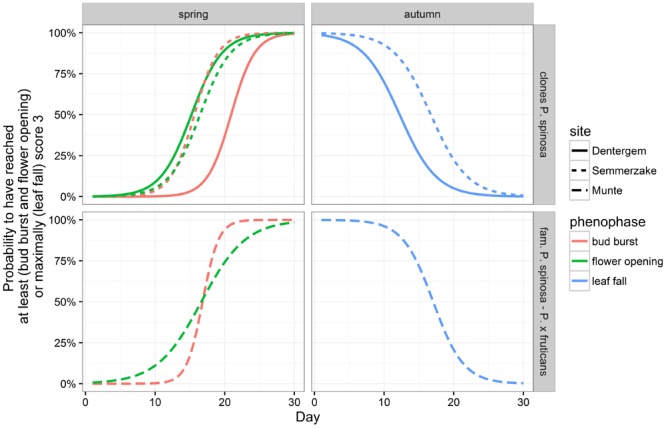
**Modeled phenological responses (bud burst, flower opening, and leaf fall) in the clone and family plantations.** Score levels for the different phenophases are in **Table 3**. Day 1 in spring is March 26 and day 1 in autumn September 23.

**FIGURE 6:**
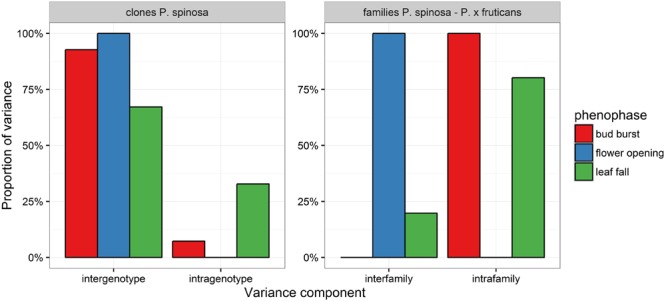
**Partitioning of variance for the individual phenophases in the clone and family plantations**.

For bud burst, flower opening and leaf fall general linear mixed models (cumulative logistic regression) were applied to visualize the phenophases (**Figure 5**). Bud burst and leaf fall were clearly dependent on the site in the clone plantations with shrubs in Dentergem flushing later in spring and senescing earlier in autumn, while flower opening did not exhibit spatial plasticity (**Figure 5**; **Table 5**). In the variance partitioning analysis, all phenophases displayed a relatively high genetic control in the clone plantations with flower opening exhibiting the strongest control, followed by bud burst and finally by leaf fall (**Figure 6**). Surprisingly, bud burst and leaf fall in the family plantation showed large relative proportions of intrafamily variation, whereas flower opening demonstrated relative large interfamily variation, suggesting a strong genetic control only for flower opening in this plantation.

**Table 5:** Model statistics for the phenological response variables in the clone plantations and the family plantation.

Phenophase	Covariate	Clone plantations				Family plantation			
		Estimate	Standard error	*z* value	*P* value	Estimate	Standard error	*z* value	*P* value
Bud burst	Day	-0.63	0.06	-10.12	**<0.001^∗∗∗^**	-0.87	0.14	-6.02	**<0.001^∗∗∗^**
	Site	-3.36	0.48	-6.97	**<0.001^∗∗∗^**				
Flower opening	Day	-0.45	0.03	-12.94	**<0.001^∗∗∗^**	-0.31	0.05	-6.62	**<0.001^∗∗∗^**
	Site	0.55	0.34	1.63	0.1				
Leaf fall	Day	0.38	0.04	8.66	**<0.001^∗∗∗^**	0.46	0.13	3.46	**<0.001^∗∗∗^**
	Site	-1.69	0.38	-4.46	**<0.001^∗∗∗^**				

*Significant results are in bold: ^∗∗∗^*P* < 0.001.*

### Trait Interrelationships

**FIGURE 7:**
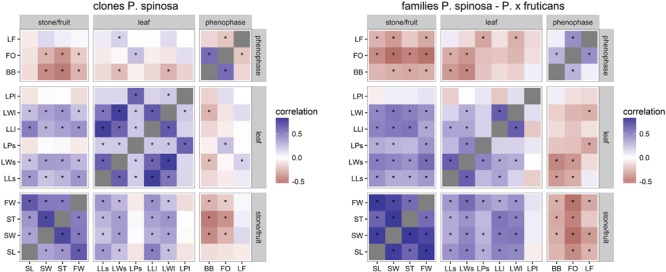
**Heat map displaying pairwise correlations between all measured and scored traits for the clone and family plantations.** Significant correlations (*P* value <0.05) are indicated with an asterisk. Trait abbreviations are in **Tables 2** and **3**.

**FIGURE 8:**
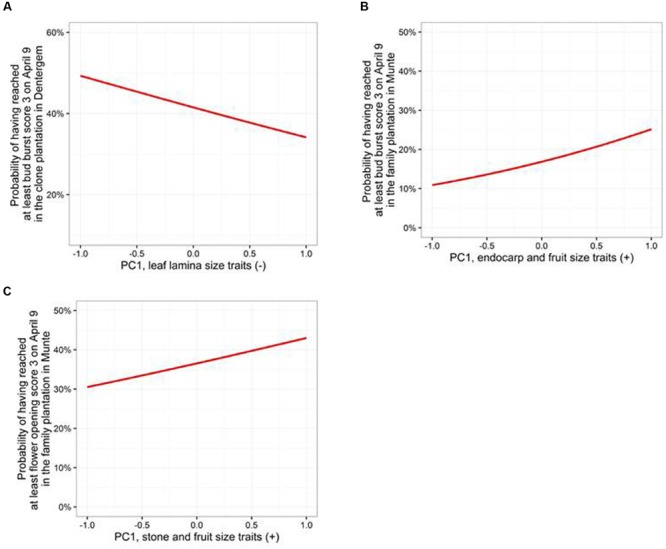
**Modeled probability of having reached a given phenological score level according to the first principal component axis in the clone and family plantations. (A)** Bud burst in the clone plantation in Dentergem. **(B)** Bud burst in the family plantation in Munte. **(C)** Flower opening in the family plantation in Munte. All probabilities were calculated based on the covariates PC2 = 0 and PC3 = 0.

Pairwise Pearson’s correlations were calculated between the traits for both the clone plantations and the family plantation (**Figure 7**). Among the morphological traits, a deviating tendency between the two types of plantations could be observed. In the clone plantation the correlations among the leaf traits and among the stone/fruit traits were more or less from the same order of magnitude, with correlations between leaf traits and endocarp/fruit traits being relatively weaker. In the family plantation the strongest correlations were found among the endocarp/fruit traits, with the correlations among the leaf traits and between leaf and endocarp/fruit traits being relatively weaker. Among the phenological traits, bud burst and flower opening correlated relatively well (+), whereas leaf fall correlated only slightly with flower opening (-) in the clone plantation. In the family plantation only a relatively minor correlation between bud burst and flower opening remained (+), whereas leaf fall did not correlate with bud burst nor flower opening. Overall, the phenological traits showed minor correlations with morphological traits. To study the relationship between morphological and phenological traits in more depth, a principal component analysis was performed on all morphological traits for the clone and the family plantations (**Table 6**). Cumulative logistic regression models were run for the three phenological traits in both the clone plantations and the family plantation including the respective three first principal component axes as covariates in the fixed part, and their explanatory power was examined. In the clone plantations only the first PC axis, mainly expressing leaf size, was significant in the bud burst model, not in the other two phenological models (**Table 7**; **Figure 8A**). In the family plantation, again only the first PC axis (mainly expressing endocarp and fruit size) was significant, although only in the bud burst and the flower opening models, not in the leaf fall model (**Table 7**; **Figures 8B,C**).

**Table 6:** Loadings of the first three principal components for the clone and family plantations.

Trait	Clones			Families		
	PC1 (44%)	PC2 (20%)	PC3 (11%)	PC1 (52%)	PC2 (13%)	PC3 (12%)
SL	-0.30	-0.30	-0.09	**0.39**	0.02	0.02
SW	-0.31	-0.24	0.50	**0.40**	0.22	0.00
ST	-0.33	-0.22	0.46	**0.37**	0.22	0.05
FWW	-0.32	-0.34	0.09	**0.38**	0.01	-0.23
LLs	**-0.37**	0.15	-0.36	0.26	-0.54	0.06
LWs	**-0.38**	0.19	-0.18	0.33	-0.39	-0.19
LPs	-0.10	0.53	0.35	0.22	-0.28	-0.44
LLl	**-0.40**	0.12	-0.30	0.31	0.12	0.48
LWl	**-0.37**	0.21	-0.13	0.30	0.39	0.19
LPl	-0.08	0.54	0.36	0.00	0.46	-0.67

*Explained variance by each axis is indicated between brackets. The four highest loadings for the first PC axes are indicated in bold. Abbreviations of traits are in **Table 2**.*

**Table 7:** Model statistics for the phenological response variables in the clone plantations and the family plantation including morphological covariates in the fixed parts of the models.

Phenophase	Covariate	Clone plantations				Family plantation			
		Estimate	Standard error	*z* value	*P* value	Estimate	Standard error	*z* value	*P* value
Bud burst	Day	-0.58	0.06	-10.17	**<0.001^∗∗∗^**	-0.88	0.15	-6.07	**<0.001^∗∗∗^**
	PC1	0.32	0.11	2.83	**0.005^∗∗^**	-0.51	0.19	-2.63	**0.009^∗∗^**
	PC2	-0.04	0.16	-0.27	0.79	0.58	0.36	1.60	0.11
	PC3	0.05	0.21	0.24	0.81	0.15	0.36	0.41	0.87
	Site	-3.19	0.46	-6.96	**<0.001^∗∗∗^**				
Flower opening	Day	-0.44	0.03	-12.92	**<0.001^∗∗∗^**	-0.33	0.05	-6.76	**<0.001^∗∗∗^**
	PC1	0.08	0.08	1.03	0.30	-0.27	0.10	-2.77	**0.006^∗∗^**
	PC2	0.11	0.13	0.91	0.36	0.09	0.17	0.55	0.58
	PC3	-0.14	0.15	-0.94	0.35	0.15	0.17	0.91	0.36
	Site	0.55	0.34	1.60	0.11				
Leaf fall	Day	0.38	0.04	8.71	**<0.001^∗∗∗^**	0.48	0.15	3.21	**0.001^∗∗^**
	PC1	-0.15	0.12	-1.27	0.20	0.47	0.43	1.09	0.28
	PC2	-0.18	0.16	-1.17	0.24	1.11	0.89	1.25	0.21
	PC3	0.03	0.21	0.15	0.88	-1.21	0.90	-1.34	0.18
	Site	-1.71	0.38	-4.49	**<0.001^∗∗∗^**				

*Significant results are in bold: ^∗∗∗^*P* < 0.001; ^∗∗^*P* < 0.01.*

## Discussion

We studied variance, genetic control and spatial phenotypic plasticity of morphological and phenological traits in *P. spinosa* and its large fruited forms. For this, we created two types of plantations: one harboring only small fruited forms (clone plantations) and one comprising both small and large fruited forms (family plantation). An exploration of the morphological data confirmed that the two types of plantations deviated in size and shape of endocarps, fruits and leaves as expected, such as more flattened and elongated endocarps and wider leaves in the large fruited forms (Depypere et al., 2009). From the mixed modeling analysis we could confirm that the large fruited forms tend to have an earlier bud burst and flower opening (Vander Mijnsbrugge et al., 2016). Several results led to new insights in the putative origin of the taxon *P. x fruticans* and to new insights in the plastic reaction of *P. spinosa* to deviating growth environments

### A Putative Hybrid Origin

Spontaneous hybrids are by no means uncommon among tree species (Stebbins, 1950; Petit and Hampe, 2006). Because of their longevity, compared to herbaceous species, selective advantage of occasional hybrids among woody plants is larger than the disadvantage of low pollen and seed fertility, which is typical for hybrids (Stebbins, 1950). An interesting phenomenon observed by Depypere et al. (2009) is a spatial genetic coherence between *P. spinosa* and *P. x fruticans* (neutral biparentally inherited AFLP markers), resulting in a stronger genetic similarity between the two taxa within a population on a specific site (intrapopulation coherence) compared to the genetic similarity within one taxon between the different studied sites (intrataxon coherence). This phenomenon was also detected, using the same molecular markers (AFLP), among related pentaploid dogrose species (De Cock et al., 2008). Although it may be tempting to search for causes in the allopolyploid genetic structure of these species groups, the explanation likely lies in the organization of the genome (both nuclear as chloroplast genomes). For the related and interfertile diploid oak species *Quercus robur* and *Quercus petraea* it has been shown that specific and more “conservative” parts of the genome are responsible for the interspecific differentiation, with “species discriminant” loci representing genome regions affected by directional selection, while the rest of the genome is “permeable” and subject to interspecific gene flow leading to common adaptations (Scotti-Saintagne et al., 2004; Neophytou et al., 2010). It is believed that this ability to preserve species discriminant traits in hybridization and backcrossing events has helped *Q. petraea* in its postglacial migration across Europe, following the footsteps of the earlier migrated *Q. robur* that displayed a more pioneering character compared to *Q. petraea* (Petit et al., 2004). For the *P. spinosa*–*P. x fruticans* group this may imply that the high interpopulation differentiation (Vander Mijnsbrugge et al., 2013; Leinemann et al., 2014), likely caused by a combination of long distance gene flow (bird dispersal) and the ability of vegetative propagation through root suckers, and additionally human disturbances of the natural populations through habitat destruction and fragmentation and through plantings with non-local stock, is imprinted in the permeable parts of the genome. However, morphological and phenological differences between *P. spinosa* and *P. x fruticans* may be preserved in the more conservative parts of the genome. Three major results from this presented analysis support this hypothesis. First, in the variance partitioning analysis of the clone plantations (small fruited *P. spinosa*), endocarp traits display the relatively strongest genetic control followed by fruit widest width, whereas in the family plantation (small and large fruited *P. spinosa*) this rank is opposite. As endocarps of both *P. spinosa* and *P. insititia* show comparable variance in size and shape characters (Depypere et al., 2007), this may indicate that in populations with a mixture of small and large fruited *P. spinosa*, the size of the fruit has a stronger diagnostic value than the endocarp traits. This is confirmed by fruit size correlating relatively stronger with endocarp length and width in the family plantation compared to the clone plantations (**Figure 7**). Accepting the hybrid origin of *P. x fruticans*, the stronger genetic control of fruit size can therefore be acknowledged as a persistent feature likely derived from the putative *P. insititia* parent and coded for in the more conservative parts of the genome. Secondly, when partitioning the variance of the three phenological traits, the largest part of the variability resided between the genotypes, and limited to zero variability remained between ramets of the same genotype in the clone plantations. This suggests strong genetic control for the phenological traits in *P. spinosa* with flower opening displaying the strongest genetic control, and leaf fall the weakest. It should be noted here that bud burst and leaf fall, but not flower opening, display spatial plasticity, which is accounted for in the fixed part of the mixed models. Surprisingly, the family plantation indicated a deviating variance pattern. Here, bud burst displayed no interfamily variation and exclusively intrafamily variation, suggesting weak genetic control, whereas the variance partitioning in flower opening was opposite, suggesting strong genetic control. Leaf fall behaved similarly to bud burst in this respect. Thus, flower opening keeps its strong genetic determinism both in the small fruited as in the mixed population (both small and large fruited), whereas for bud burst and leaf fall in the mixed population the genetic control is lessened. Accepting *P. x fruticans* as originating from a hybrid cross with the cultivated *P. insititia* followed by backcrossing with the *P. spinosa* parent, the *P. insititia* parent may have distorted the mechanisms leading to the timing of bud burst and leaf fall, seemingly without clear selective disadvantage since many natural populations of *P. spinosa* in the northern part of Belgium contain small and large fruited forms (Vander Mijnsbrugge et al., 2013). Possibly, this may have caused the different rank order between flushing and flowering in a provenance trial, with provenances of *P. spinosa* containing large fruited forms, although both phenophases occur congruently (Vander Mijnsbrugge et al., 2016). Alternatively, as we compare a clone experiment with a half-sib offspring experiment, the unknown pollen donors in the family experiment may have influenced these results. Thirdly, pubescence on the central vein at the lower side of the leaf lamina is an intriguing trait. Whereas this hairiness is analogously distributed for short shoot and long shoot leaves in the clone plantations and for the short shoot leaves in the family plantation, it surprisingly deviates for exposed long shoot leaves in the family plantation where it is more abundantly present (**Figure 3**). As *P. insititia* is known for pubescent undersides of leaves (Scholtz and Scholtz, 1995), and accepting *P. x fruticans* as descendants of a hybridization process between *P. spinosa* and *P. insititia*, the distribution pattern of pubescence on the central vein of long shoot leaves in the family plantation can be derived from the *P. insititia* parent. Still, in the family plantation pubescence on long shoot leaves does not correlate with endocarp, fruit, and other leaf traits. This may imply that dense leaf pubescence from *P. insititia* long shoot leaves entered the *P. spinosa* populations many generations ago and the expected correlation between this pubescence and size traits of endocarp, fruit, and leaves may have faded through many cycles of recombination in which the specific combination of size traits and pubescence was not selectively advantageous. As pubescence is generally supposed to protect against dehydration and evolved specifically in more arid regions (Büsgen et al., 1929), this leaf pubescence may have remained on the long shoot leaves of mixed populations (*P. spinosa*–*P. x fruticans*) with the long shoots being more exposed to extreme weather conditions compared to the short shoot leaves.

### Spatial Phenotypic Plasticity

Being able to shift a phenotype in correspondence to changes in the environment allows an individual to maintain its fitness (Nonaka et al., 2015). Plasticity is of particular importance for trees as they are characterized by long generation times and, consequently, experience substantial variation in growth conditions throughout their lifetime (Valladares et al., 2007; Nicotra et al., 2010). The study of plasticity in trees may help our comprehension of how trees will cope with the predicted climate change. Comparing ramets at the two clone plantation sites, three major results can be stressed from our spatial plasticity analysis. Firstly, among the observed phenophases flower opening remarkably does not demonstrate any spatial plasticity between the two plantations, whereas bud burst is delayed and leaf fall is advanced in Dentergem compared to Semmerzake. As plasticity comes at a certain cost (Auld et al., 2010), it can be hypothesized that reduced fecundity due to flower opening not being able to track exceptional unfavorable conditions in *P. spinosa* is less detrimental than injurious vegetative bud burst hampering the start of the growing season. Second, the more exposed and thus less favorable conditions in Dentergem led to a shorter growing season. Fruit size was reduced in these less advantageous growing conditions, whereas leaf width tended to enlarge, leading to deviating responses of different organs to the heterogeneous growing conditions in the two plantation sites. As the plantation in Dentergem is more exposed compared to Semmerzake, it can be hypothesized that enlarging the surface of the leaf blade more in comparison to the lengthening of the leaf margin is a protection against strong impacting winds that may cause too strong evaporation. It is well known that a smaller leaf margin/leaf area ratio reduces the evaporation of the leaf (Büsgen et al., 1929). Thirdly, in the clone plantations the plastic variance caused by the different sites (site as covariate in the fixed part of the model) is separated from the intergenotype and intragenotype variance (random part of the model). This can lead to additional insights in morphological variability. In contrast to the larger spatial plasticity of length and width of long shoot leaves relative to short shoot leaves (clone plantations, *P. spinosa*), the variance partitioning does not suggest a stronger genetic control for these traits on short shoot leaves compared to long shoot leaves (clone plantations, *P. spinosa* and family plantation, *P. spinosa*–*P. x fruticans*), implying that size and shape of long shoot leaves react plastically on deviating growing conditions but are similarly genetically determined in similar conditions. Although traditionally long shoot leaves are neglected in taxonomical issues within the genus *Prunus*, being considered as too variable (Maes, 2013), our results indicate that long shoot leaves can have a noteworthy taxonomic value when growing conditions are similar.

## Conclusion

Although genetic analyses have suggested the classification of *P. x fruticans* as a large fruited form of *P. spinosa* (Depypere et al., 2009; Vander Mijnsbrugge et al., 2013), recent insights in genome structuring of hybrids between related sympatric and interfertile woody species, identifying permeable and conservative parts, together with several of the here presented results on morphological and phenological variability, advocate the origin of *P. x fruticans* as a historical hybrid between *P. spinosa* and *P. insititia*, followed by subsequent backcrossings with the *P. spinosa* parent. This implies a crop-to-wild gene flow, possibly dating back to the introduction of *P. insititia* as a fruit tree, which is suggested to have occurred already in Neolithic times in central Europe (Körber-Grohne, 1996), with no indications yet for negative consequences for *P. spinosa*. Some plastic reactions of *P. spinosa* may help the shrub to cope with the predicted climate change, such as the enlarging of the ratio leaf area/leaf margin as a response to drought.

## Author Contributions

KV and LD defined the research, collected seeds and cuttings, organized the growth of planting stock, and designed and planted the experimental plantations. KV, LD, AT, and MS organized the measurements and observations in the experimental plantations, and helped with the statistical analysis. KV and AT wrote the manuscript. MS supervised the whole project.

## Conflict of Interest Statement

The authors declare that the research was conducted in the absence of any commercial or financial relationships that could be construed as a potential conflict of interest.
