# Supplementary material for: Variance, Genetic Control, and Spatial Phenotypic Plasticity of Morphological and Phenological Traits in Prunus spinosa and Its Large Fruited Forms (P. x fruticans)
Source: Front Plant Sci. 2016 Nov 3;7:1641. doi: 10.3389/fpls.2016.01641 (PMC5093327; doi:10.3389/fpls.2016.01641)
Supplement: Supplementary file 1 [file Table_1.DOCX]

Supplementary Material

**Variance, genetic control and spatial phenotypic plasticity of morphological and phenological traits in *Prunus* *spinosa* and its large fruited forms *(P. x fruticans)***

**Kristine Vander Mijnsbrugge^1*^, Arion Turcsán^1,2,3^, Leander Depypere^4^, Marijke Steenackers**^1^

^1^ Department of Forest Genetic Resources, Research Institute for Nature and Forest, Geraardsbergen, Belgium

^2^ Department of Biometrics and Agricultural Informatics, Szent István University, Budapest, Hungary

^3^ Department of Forest Reproductive Material and Plantation Management, West-Hungarian University, Institute of Silviculture and Forest Protection, Sopron, Hungary

^4^ Groeningenlaan 70, 8500 Kortrijk, Belgium

**^*^Correspondence:**

Dr. ir. Kristine Vander Mijnsbrugge (first name: Kristine, last name: Vander Mijnsbrugge)

[kristine.vandermijnsbrugge@inbo.be](mailto:kristine.vandermijnsbrugge@inbo.be)

## Supplementary Tables

**Supplementary Table S1.** Basic measurements and observations for the plants in the clone plantations of Dentergem and Semmerzake. Abbreviations of traits are in Tables 2 and 3.

| Loc* | plantID | cloneID | SL (mm) | SW (mm) | ST (mm) | FWW (mm) | LLs (mm) | LWs (mm) | LPs | LLl (mm) | LWl (mm) | LPl | BB 26ma | FO 26ma | BB 1ap | FO 1ap | BB 9ap | FO 9ap | BB 16ap | FO 16ap | BB 20ap | FO 20ap | FA 29se | FA 13oc |
| --- | --- | --- | --- | --- | --- | --- | --- | --- | --- | --- | --- | --- | --- | --- | --- | --- | --- | --- | --- | --- | --- | --- | --- | --- |
| S | 64 | 1 | 9,6 | 8,2 | 6,55 | 15,3 | 50 | 22 | 2 | 47 | 23 | 2 | 2 | 1 |  |  | 2 | 2 | 4 | 6 |  |  | 1 | 2 |
| S | 68 | 1 | 8,8 | 7,65 | 6,15 | 13,6 | 41 | 17 | 2 | 46 | 19 | 2 | 1 | 1 |  |  | 2 | 2 | 4 | 6 |  |  | 1 | 3 |
| D | 91 | 1 | 7,9 | 9,25 | 6,7 | 14,3 | 38 | 17 | 1 | 39 | 17 | 1 |  |  | 1 | 2 |  |  |  |  | 4 | 6 | 2 | 2 |
| D | 115 | 1 | 9,5 | 8,5 | 6,65 | 13,9 | 40 | 21 | 3 | 43 | 21 | 2 |  |  | 1 | 2 |  |  |  |  | 4 | 6 | 2 | 2 |
| S | 36 | 3 | 10,5 | 8,7 | 6,6 | 14,9 | 39 | 16 | 1 | 41 | 18 | 2 | 1 | 2 |  |  | 1 | 2 | 4 | 6 |  |  | 4 | 5 |
| S | 44 | 3 | 9,75 | 8,05 | 6,2 | 13,8 | 32 | 14 | 1 | 33 | 14 | 1 | 1 | 2 |  |  | 2 | 2 | 4 | 6 |  |  | 2 | 4 |
| S | 25 | 6 | 7,7 | 6,95 | 5,85 | 12,3 | 30 | 13 | 3 | 36 | 15 | 2 | 1 | 2 |  |  | 2 | 3 | 4 | 6 |  |  | 1 | 2 |
| S | 38 | 6 | 8,2 | 7,05 | 5,7 | 13,4 | 37 | 17 | 2 | 37 | 18 | 2 | 2 | 2 |  |  | 2 | 3 | 5 | 6 |  |  | 1 | 2 |
| D | 90 | 6 | 7,6 | 6,75 | 5,95 | 11,6 | 35 | 16 | 3 | 38 | 21 | 3 |  |  | 1 | 2 |  |  |  |  | 4 | 6 | 2 | 3 |
| S | 47 | 8 | 8,65 | 6,75 | 5,25 | 12,1 | 41 | 18 | 2 | 41 | 18 | 2 | 1 | 1 |  |  | 4 | 2 | 4 | 5 |  |  | 2 | 4 |
| S | 53 | 8 | 9,5 | 6,9 | 5,1 | 12,9 | 39 | 16 | 2 | 41 | 18 | 1 | 1 | 2 |  |  | 2 | 2 | 5 | 6 |  |  | 2 | 4 |
| S | 59 | 8 | 9,1 | 6,55 | 5 | 12,8 | 42 | 19 | 2 | 42 | 19 | 2 | 1 | 2 |  |  | 2 | 2 | 4 | 5 |  |  | 1 | 4 |
| S | 76 | 8 | 8,65 | 6,65 | 5 | 12,6 | 35 | 15 | 2 | 40 | 16 | 2 | 2 | 1 |  |  | 3 | 2 | 5 | 5 |  |  | 1 | 3 |
| S | 79 | 8 | 8,55 | 6,5 | 4,95 | 12,1 | 26 | 13 | 2 | 32 | 14 | 2 | 1 | 1 |  |  | 2 | 2 | 4 | 5 |  |  | 2 | 4 |
| D | 83 | 8 | 8,9 | 7,1 | 5,35 | 13,1 | 27 | 13 | 1 | 35 | 14 | 1 |  |  | 1 | 2 |  |  |  |  | 4 | 5 | 3 | 4 |
| D | 92 | 8 | 9,9 | 7,15 | 5,45 | 12,6 | 32 | 15 | 2 | 39 | 16 | 2 |  |  | 1 | 2 |  |  |  |  | 3 | 5 | 4 | 4 |
| D | 208 | 8 | 9,5 | 7,45 | 5,85 | 13 | 33 | 13 | 1 | 42 | 20 | 1 |  |  | 1 | 1 |  |  |  |  | 4 | 6 | 3 | 4 |
| D | 216 | 8 |  |  |  |  | 40 | 16 | 3 | 41 | 19 | 2 |  |  | 2 | 2 |  |  |  |  | 4 | 6 | 2 | 2 |
| S | 20 | 9 | 11,5 | 8,3 | 6,35 | 14,7 | 41 | 15 | 2 | 50 | 18 | 1 | 1 | 1 |  |  | 1 | 2 | 3 | 6 |  |  | 2 | 4 |
| S | 42 | 9 | 10,8 | 8,25 | 6,15 | 14,7 | 36 | 16 | 2 | 51 | 20 | 2 | 1 | 2 |  |  | 2 | 2 | 4 | 6 |  |  | 2 | 4 |
| S | 51 | 9 | 10,4 | 7,55 | 5,8 | 13,4 | 40 | 16 | 1 | 54 | 20 | 1 | 1 | 2 |  |  | 2 | 3 | 4 | 6 |  |  | 1 | 3 |
| S | 19 | 10 | 9,1 | 7,55 | 6 | 12,7 | 29 | 13 | 1 | 34 | 15 | 1 | 1 | 1 |  |  | 3 | 3 | 4 | 5 |  |  | 1 | 3 |
| D | 86 | 10 | 8,65 | 7,75 | 6,1 | 12,3 | 40 | 16 | 2 | 41 | 17 | 2 |  |  | 1 | 2 |  |  |  |  | 4 | 7 | 2 | 3 |
| D | 106 | 10 | 8,7 | 7,9 | 6,4 | 12,6 | 35 | 13 | 1 | 35 | 14 | 1 |  |  | 1 | 2 |  |  |  |  | 4 | 7 | 1 | 3 |
| S | 24 | 11 | 9,4 | 7,25 | 5,05 | 14,4 | 36 | 14 | 1 | 39 | 15 | 1 | 1 | 2 |  |  | 2 | 2 | 4 | 6 |  |  | 1 | 2 |
| S | 33 | 11 | 9,75 | 7,3 | 5,6 | 14,4 | 35 | 14 | 1 | 40 | 15 | 1 | 1 | 3 |  |  | 2 | 3 | 4 | 6 |  |  | 1 | 2 |
| S | 48 | 11 | 9,4 | 8,1 | 5,75 | 14,5 | 46 | 18 | 1 | 48 | 21 | 1 | 2 | 2 |  |  | 4 | 3 | 5 | 6 |  |  | 1 | 2 |
| S | 75 | 11 | 10,2 | 7,45 | 5,25 | 15 | 42 | 17 | 1 | 47 | 17 | 1 | 2 | 2 |  |  | 3 | 3 | 4 | 6 |  |  | 1 | 4 |
| D | 203 | 11 | 9,45 | 7,6 | 5,4 | 13 | 39 | 18 | 1 | 45 | 19 | 1 |  |  | 2 | 2 |  |  |  |  | 4 | 6 | 2 | 3 |
| D | 217 | 11 | 9,75 | 7,95 | 5,25 | 12,9 | 28 | 11 | 1 | 36 | 15 | 2 |  |  | 2 | 2 |  |  |  |  | 4 | 6 | 2 | 3 |
| S | 28 | 12 | 8,55 | 9,3 | 6,95 | 15,4 | 38 | 17 | 2 | 40 | 18 | 2 | 3 | 2 |  |  | 5 | 3 | 6 | 6 |  |  | 1 | 4 |
| S | 35 | 12 | 8,55 | 8,8 | 6,65 | 13 | 43 | 20 | 2 | 43 | 20 | 2 | 3 | 2 |  |  | 5 | 4 | 6 | 6 |  |  | 1 | 3 |
| S | 78 | 12 | 8,95 | 9,2 | 7,1 | 14,4 | 41 | 18 | 3 | 52 | 24 | 3 | 3 | 4 |  |  | 5 | 4 | 6 | 6 |  |  | 1 | 3 |
| D | 202 | 12 | 8,25 | 8,7 | 6,5 | 12,2 | 35 | 14 | 1 | 41 | 16 | 1 |  |  | 3 | 3 |  |  |  |  | 5 | 7 | 2 | 3 |
| D | 102 | 12 | 8,15 | 9,05 | 6,7 | 15,4 | 38 | 17 | 3 | 40 | 19 | 2 |  |  | 2 | 3 |  |  |  |  | 6 | 7 | 2 | 3 |
| S | 10 | 13 | 8,8 | 6,85 | 6,15 | 12,5 | 35 | 14 | 1 | 38 | 16 | 2 | 2 | 2 |  |  | 4 | 3 | 5 | 6 |  |  | 2 | 4 |
| S | 58 | 13 | 9,1 | 7,1 | 6,1 | 12,7 | 39 | 13 | 2 | 45 | 20 | 3 | 2 | 2 |  |  | 4 | 3 | 6 | 6 |  |  | 1 | 3 |
| D | 99 | 13 | 9,4 | 7,3 | 6,3 | 13,2 | 35 | 14 | 2 | 42 | 17 | 3 |  |  | 2 | 2 |  |  |  |  | 5 | 7 | 3 | 3 |
| D | 215 | 13 | 9,75 | 7,2 | 5,75 | 9,65 | 40 | 17 | 3 | 48 | 21 | 2 |  |  |  |  |  |  |  |  |  |  | 2 | 3 |
| S | 32 | 14 | 8,3 | 7,7 | 5,6 | 12,6 | 36 | 15 | 1 | 40 | 17 | 1 | 2 | 2 |  |  | 3 | 3 | 5 | 6 |  |  | 1 | 2 |
| S | 49 | 14 | 8 | 7,2 | 5,25 | 11,7 | 38 | 16 | 1 | 38 | 16 | 1 | 1 | 1 |  |  | 3 | 2 | 5 | 6 |  |  | 1 | 2 |
| S | 52 | 14 | 8,6 | 7,6 | 5,3 | 12 | 33 | 15 | 1 | 37 | 15 | 1 | 2 | 2 |  |  | 3 | 3 | 5 | 6 |  |  | 1 | 3 |
| S | 54 | 14 | 8,9 | 7,9 | 5,65 | 14,6 | 40 | 17 | 1 | 44 | 19 | 1 | 1 | 1 |  |  | 3 | 3 | 5 | 6 |  |  | 2 | 3 |
| S | 67 | 14 | 8,2 | 7,25 | 5,4 | 12,9 | 36 | 14 | 1 | 38 | 15 | 1 | 2 | 1 |  |  | 3 | 2 | 5 | 6 |  |  | 2 | 3 |
| S | 74 | 14 | 8,6 | 7,35 | 5,4 | 12,5 | 31 | 14 | 1 | 39 | 15 | 1 | 2 | 2 |  |  | 3 | 2 | 4 | 6 |  |  | 1 | 2 |
| D | 84 | 14 | 8,85 | 8 | 5,95 | 13,4 | 30 | 11 | 1 | 34 | 15 | 1 |  |  | 1 | 2 |  |  |  |  | 4 | 6 | 2 | 3 |
| D | 88 | 14 | 8,75 | 8,15 | 6 | 13 | 34 | 14 | 2 | 38 | 15 | 1 |  |  | 1 | 1 |  |  |  |  | 4 | 6 | 2 | 3 |
| D | 93 | 14 | 8,75 | 8,2 | 6,05 | 12,5 | 32 | 13 | 1 | 37 | 14 | 1 |  |  | 1 | 2 |  |  |  |  | 4 | 6 | 2 | 2 |
| D | 105 | 14 | 10,1 | 8,3 | 6,3 | 14,2 | 60 | 18 | 3 | 60 | 22 | 3 |  |  | 1 | 2 |  |  |  |  | 4 | 6 | 3 | 3 |
| S | 9 | 15 | 10,5 | 7,85 | 6,15 | 14,9 | 38 | 18 | 1 | 50 | 22 | 1 | 2 | 1 |  |  | 3 | 2 | 5 | 6 |  |  | 2 | 3 |
| S | 22 | 15 | 9,75 | 6,5 | 5,35 | 13,6 | 43 | 17 | 1 | 46 | 18 | 1 | 2 | 1 |  |  | 2 | 3 | 5 | 5 |  |  | 1 | 3 |
| S | 45 | 15 | 8,7 | 7 | 5,95 | 12,6 | 35 | 13 | 1 | 32 | 14 | 1 | 1 | 2 |  |  | 3 | 4 | 4 | 6 |  |  | 3 | 3 |
| D | 207 | 15 | 10,3 | 7,85 | 6,6 | 13,6 | 35 | 15 | 1 | 38 | 15 | 1 |  |  | 2 | 3 |  |  |  |  | 5 | 6 | 2 | 3 |
| D | 103 | 15 | 8,75 | 7,85 | 5,9 | 11,9 | 37 | 17 | 1 | 40 | 20 | 1 |  |  | 1 | 3 |  |  |  |  | 4 | 6 | 2 | 3 |
| S | 16 | 16 | 9,9 | 6,75 | 5,4 | 14 | 35 | 13 | 1 | 34 | 17 | 1 | 2 | 1 |  |  | 3 | 3 | 4 | 6 |  |  | 1 | 3 |
| S | 17 | 16 | 10,1 | 7,65 | 6,4 | 14,4 | 41 | 14 | 1 | 48 | 16 | 1 | 1 | 1 |  |  | 2 | 3 | 4 | 6 |  |  | 1 | 3 |
| S | 71 | 16 | 10,8 | 7,8 | 6,2 | 13,2 | 45 | 15 | 1 | 44 | 16 | 1 | 2 | 3 |  |  | 3 | 4 | 5 | 6 |  |  | 1 | 3 |
| S | 81 | 16 | 10,8 | 8,25 | 6,75 | 15,7 | 40 | 16 | 1 | 45 | 16 | 1 | 1 | 3 |  |  | 3 | 3 | 5 | 6 |  |  | 1 | 2 |
| D | 96 | 16 | 9,85 | 7,6 | 6,1 | 14,6 | 40 | 15 | 1 | 40 | 16 | 1 |  |  | 2 | 3 |  |  |  |  | 5 | 6 | 2 | 3 |
| D | 109 | 16 | 9,8 | 7,8 | 6,3 | 13,6 | 45 | 17 | 1 | 50 | 18 | 2 |  |  | 1 | 2 |  |  |  |  | 4 | 7 | 2 | 3 |
| D | 218 | 16 | 10,5 | 7,25 | 6,1 | 13,7 | 43 | 16 | 1 | 50 | 18 | 1 |  |  | 1 | 2 |  |  |  |  | 5 | 6 | 2 | 2 |
| S | 21 | 18 | 9,05 | 7,55 | 5,8 | 12,4 | 30 | 13 | 3 | 32 | 14 | 3 | 1 | 1 |  |  | 2 | 2 | 5 | 5 |  |  | 1 | 3 |
| S | 50 | 18 | 9,7 | 7,8 | 5,7 | 14,1 | 37 | 15 | 3 | 40 | 16 | 4 | 1 | 2 |  |  | 2 | 2 | 5 | 6 |  |  | 1 | 2 |
| S | 65 | 18 | 8,45 | 6,7 | 5,1 | 13,1 | 40 | 15 | 3 | 41 | 16 | 4 | 1 | 1 |  |  | 2 | 2 | 5 | 5 |  |  | 1 | 3 |
| D | 210 | 18 | 9 | 7,6 | 5,65 | 12 | 35 | 16 | 2 | 35 | 16 | 2 |  |  | 1 | 1 |  |  |  |  | 4 | 6 | 3 | 3 |
| S | 201 | 19 |  |  |  |  |  |  |  |  |  |  | 2 | 2 |  |  | 3 | 4 | 4 | 6 |  |  | 1 | 3 |
| S | 30 | 19 | 9,7 | 8,2 | 6,15 | 13,3 | 32 | 15 | 1 | 32 | 17 | 1 | 2 | 2 |  |  | 2 | 4 | 4 | 6 |  |  | 1 | 3 |
| S | 57 | 19 | 12,2 | 8 | 6,8 | 16,8 | 44 | 13 | 1 | 49 | 14 | 1 | 3 | 2 |  |  | 4 | 4 | 5 | 6 |  |  | 1 | 3 |
| D | 110 | 19 | 9,45 | 8 | 6,15 | 13 | 42 | 19 | 1 | 41 | 20 | 1 |  |  | 1 | 2 |  |  |  |  | 4 | 6 | 2 | 3 |
| D | 213 | 19 |  |  |  |  | 44 | 20 | 1 | 48 | 22 | 1 |  |  |  |  |  |  |  |  |  |  | 2 | 2 |
| S | 2 | 20 | 9,1 | 7,7 | 5,7 | 13,1 | 28 | 12 | 1 | 33 | 14 | 1 | 1 | 3 |  |  | 3 | 4 | 5 | 6 |  |  | 1 | 3 |
| S | 5 | 20 | 9 | 7,5 | 5,55 | 12,9 | 30 | 14 | 1 | 32 | 15 | 1 | 2 | 1 |  |  | 4 | 4 | 4 | 6 |  |  | 1 | 2 |
| S | 6 | 20 | 9,5 | 8 | 5,75 | 12,6 | 30 | 13 | 1 | 30 | 14 | 1 | 1 | 1 |  |  | 2 | 4 | 5 | 6 |  |  | 1 | 3 |
| S | 8 | 20 | 9,7 | 7,6 | 5,75 | 12,4 | 28 | 13 | 1 | 30 | 15 | 1 | 2 | 1 |  |  | 3 | 4 | 5 | 6 |  |  | 1 | 3 |
| D | 204 | 20 | 9,05 | 8,1 | 6 | 11,8 | 43 | 20 | 1 | 44 | 22 | 1 |  |  | 2 | 3 |  |  |  |  | 5 | 7 | 2 | 3 |
| D | 104 | 20 | 8,5 | 8,4 | 6 | 12,6 | 36 | 14 | 1 | 37 | 15 | 1 |  |  | 1 | 1 |  |  |  |  | 4 | 6 | 2 | 3 |
| D | 214 | 20 | 9,2 | 7,7 | 5,65 | 12 | 35 | 18 | 1 | 35 | 18 | 2 |  |  |  |  |  |  |  |  |  |  | 2 | 2 |
| S | 29 | 22 | 7,45 | 6,65 | 5,35 | 11,1 | 29 | 13 | 1 | 31 | 14 | 1 | 1 | 1 |  |  | 2 | 2 | 4 | 6 |  |  | 1 | 2 |
| S | 56 | 22 | 7,3 | 6,4 | 5,15 | 10,9 | 27 | 13 | 2 | 30 | 14 | 1 | 1 | 2 |  |  | 2 | 2 | 4 | 6 |  |  | 1 | 3 |
| S | 69 | 22 | 7,35 | 6,25 | 5,1 | 10,4 | 29 | 14 | 1 | 32 | 15 | 1 | 1 | 2 |  |  | 2 | 2 | 4 | 6 |  |  | 1 | 4 |
| D | 107 | 22 | 7,3 | 7,6 | 5,7 | 11,3 | 31 | 16 | 1 | 40 | 19 | 1 |  |  | 1 | 2 |  |  |  |  | 4 | 6 | 3 | 4 |
| S | 60 | 23 | 8,65 | 7,15 | 5,95 | 12,4 | 31 | 12 | 1 | 35 | 15 | 1 | 2 | 2 |  |  | 3 | 3 | 4 | 6 |  |  | 1 | 4 |
| S | 62 | 23 | 9,35 | 7,5 | 6 | 14 | 37 | 14 | 1 | 45 | 16 | 1 | 1 | 2 |  |  | 2 | 3 | 4 | 6 |  |  | 2 | 5 |
| S | 66 | 23 | 8,7 | 7,2 | 5,95 | 12,8 | 42 | 17 | 1 | 44 | 17 | 1 | 1 | 2 |  |  | 3 | 4 | 4 | 6 |  |  | 2 | 5 |
| D | 205 | 23 | 8,8 | 7,4 | 5,9 | 11 | 35 | 18 | 2 | 41 | 18 | 1 |  |  | 1 | 3 |  |  |  |  | 4 | 6 | 2 | 3 |
| D | 119 | 23 | 8,75 | 7,25 | 6 | 11,3 | 38 | 17 | 1 | 40 | 18 | 1 |  |  | 1 | 2 |  |  |  |  | 4 | 6 | 2 | 3 |
| S | 3 | 24 | 8,25 | 6,75 | 5,6 | 12,4 | 35 | 13 | 1 | 33 | 14 | 1 | 1 | 2 |  |  | 2 | 3 | 4 | 6 |  |  | 1 | 3 |
| S | 37 | 24 | 9,45 | 7,75 | 6,35 | 13,7 | 37 | 14 | 1 | 40 | 18 | 1 | 1 | 2 |  |  | 2 | 3 | 4 | 6 |  |  | 1 | 3 |
| S | 39 | 24 | 9,25 | 7,65 | 6 | 13,9 | 42 | 17 | 1 | 41 | 18 | 1 | 1 | 1 |  |  | 2 | 3 | 4 | 6 |  |  | 1 | 3 |
| S | 46 | 24 | 9,35 | 7,55 | 6,25 | 13,5 | 35 | 15 | 1 | 36 | 16 | 1 | 1 | 2 |  |  | 2 | 3 | 4 | 6 |  |  | 1 | 3 |
| S | 73 | 24 | 8,5 | 6,95 | 5,5 | 12,6 | 32 | 11 | 1 | 31 | 19 | 1 | 1 | 3 |  |  | 3 | 3 | 4 | 6 |  |  | 2 | 3 |
| D | 85 | 24 | 8,85 | 7,05 | 5,7 | 13 | 46 | 18 | 1 | 50 | 20 | 1 |  |  | 1 | 2 |  |  |  |  | 4 | 6 | 2 | 4 |
| D | 87 | 24 | 8,8 | 7,15 | 6 | 13,3 | 34 | 15 | 1 | 42 | 18 | 1 |  |  | 1 | 2 |  |  |  |  | 4 | 6 | 3 | 4 |
| D | 108 | 24 | 9,6 | 7,7 | 6,05 | 12,3 | 28 | 11 | 2 | 41 | 17 | 2 |  |  | 1 | 2 |  |  |  |  | 4 | 6 | 1 | 3 |
| S | 11 | 25 | 10,1 | 9,15 | 7,5 | 15,2 | 39 | 19 | 1 | 44 | 21 | 1 | 2 | 2 |  |  | 4 | 3 | 6 | 6 |  |  | 1 | 3 |
| S | 18 | 25 | 9,95 | 9,25 | 7,55 | 14,3 | 37 | 20 | 1 | 48 | 25 | 2 | 3 |  |  |  | 4 | 3 | 6 | 6 |  |  | 1 | 3 |
| S | 34 | 25 | 10,1 | 9,3 | 7,2 | 14,2 | 42 | 23 | 1 | 50 | 23 | 3 | 3 | 3 |  |  | 4 | 3 | 5 | 6 |  |  | 1 | 3 |
| D | 89 | 25 | 9,9 | 8,6 | 7,55 | 15 | 35 | 20 | 1 | 38 | 20 | 1 |  |  | 2 | 2 |  |  |  |  | 5 | 6 | 2 | 3 |
| D | 120 | 25 | 10,2 | 8,9 | 7,6 | 14,2 | 36 | 20 | 2 | 49 | 25 | 1 |  |  | 2 | 3 |  |  |  |  | 6 | 7 | 2 | 3 |
| S | 7 | 27 | 8,45 | 7,8 | 5,95 | 12,1 | 31 | 13 | 1 | 34 | 15 | 2 | 1 | 1 |  |  | 2 | 2 | 4 | 6 |  |  | 1 | 3 |
| S | 12 | 27 | 8,95 | 8,25 | 6 | 13,3 | 40 | 18 | 2 | 42 | 18 | 1 | 1 | 1 |  |  | 2 | 2 | 4 | 6 |  |  | 2 | 4 |
| S | 14 | 27 | 8,25 | 8,05 | 5,75 | 12,4 | 36 | 15 | 1 | 39 | 17 | 2 | 1 | 1 |  |  | 2 | 2 | 4 | 5 |  |  | 1 | 3 |
| S | 40 | 27 | 8,5 | 8,05 | 5,95 | 13,3 | 31 | 13 | 2 | 32 | 13 | 1 | 1 | 2 |  |  | 2 | 2 | 4 | 6 |  |  | 2 | 4 |
| D | 206 | 27 | 8 | 7,4 | 5,45 | 10,4 | 35 | 16 | 3 | 40 | 18 | 3 |  |  | 1 | 1 |  |  |  |  | 4 | 6 | 2 | 3 |
| D | 209 | 27 | 9 | 8,2 | 6,15 | 12,2 | 26 | 14 | 1 | 40 | 18 | 1 |  |  | 1 | 1 |  |  |  |  | 4 | 6 | 2 | 3 |
| S | 15 | 28 | 7,7 | 7,45 | 5,5 | 11,1 | 34 | 13 | 1 | 34 | 13 | 2 | 1 | 2 |  |  | 3 | 3 | 4 | 6 |  |  | 2 | 3 |
| S | 61 | 28 | 7,85 | 7,15 | 5,5 | 11,3 | 33 | 12 | 1 | 36 | 14 | 3 | 2 | 3 |  |  | 3 | 4 | 4 | 6 |  |  | 2 | 4 |
| D | 94 | 28 | 7,4 | 6,65 | 5,35 | 11,8 | 30 | 13 | 2 | 42 | 15 | 2 |  |  | 1 | 3 |  |  |  |  | 4 | 6 | 3 | 3 |
| S | 31 | 29 | 10,6 | 8,3 | 5,95 | 15 | 46 | 20 | 2 | 54 | 20 | 2 | 1 | 1 |  |  | 2 | 2 | 4 | 5 |  |  | 2 | 3 |
| S | 55 | 29 | 10,2 | 8,3 | 6,15 | 15,2 | 57 | 20 | 2 | 55 | 21 | 1 | 1 | 2 |  |  | 2 | 3 | 4 | 5 |  |  | 1 | 2 |
| D | 114 | 29 | 10,4 | 8,05 | 6,35 | 13,8 | 58 | 25 | 2 | 72 | 28 | 2 |  |  | 2 | 2 |  |  |  |  | 4 | 6 | 2 | 3 |
| S | 23 | 30 | 10,8 | 8,4 | 6,05 | 14,1 | 24 | 12 | 1 | 31 | 14 | 1 | 2 | 2 |  |  | 3 | 3 | 5 | 6 |  |  | 1 | 3 |
| S | 63 | 30 | 11,2 | 8,65 | 6,15 | 16,6 | 36 | 17 | 1 | 44 | 19 | 1 | 2 | 3 |  |  | 4 | 3 | 5 | 6 |  |  | 3 | 5 |
| D | 100 | 30 | 9,4 | 7,5 | 6,2 | 13,6 | 36 | 17 | 1 | 40 | 20 | 1 |  |  | 2 | 2 |  |  |  |  | 4 | 6 | 2 | 3 |
| S | 70 | 31 | 8,6 | 8,1 | 5,5 | 12,3 | 41 | 17 | 3 | 45 | 18 | 2 | 3 | 2 |  |  | 4 | 4 | 5 | 6 |  |  | 1 | 3 |
| S | 82 | 31 | 8,35 | 7,6 | 5,4 | 12,5 | 31 | 12 | 1 | 37 | 15 | 1 | 1 | 2 |  |  | 2 | 3 | 5 | 6 |  |  | 1 | 2 |
| D | 101 | 31 | 8,05 | 7,25 | 5,45 | 12,2 | 42 | 16 | 1 | 34 | 18 | 2 |  |  | 2 | 2 |  |  |  |  | 4 | 7 | 2 | 4 |
| S | 4 | 33 | 9,35 | 7,25 | 5,45 | 13,2 | 30 | 15 | 1 | 34 | 14 | 2 | 1 | 2 |  |  | 2 | 3 | 5 | 6 |  |  | 1 | 2 |
| S | 77 | 33 | 9,7 | 8,15 | 6 | 14,3 | 40 | 21 | 1 | 46 | 22 | 2 | 3 | 2 |  |  | 4 | 3 | 5 | 6 |  |  | 1 | 2 |
| D | 97 | 33 | 9,2 | 7,1 | 5,5 | 12,8 | 38 | 19 | 1 | 43 | 24 | 1 |  |  | 1 | 2 |  |  |  |  | 6 | 6 | 1 | 2 |
| D | 212 | 33 | 9,05 | 7,4 | 5,85 | 10,9 | 43 | 19 | 2 | 47 | 23 | 4 |  |  |  |  |  |  |  |  |  |  | 2 | 2 |
| S | 80 | 34 | 8,6 | 6,85 | 5,5 | 11,6 | 32 | 12 | 1 | 37 | 15 | 2 | 2 | 2 |  |  | 4 | 3 | 5 | 6 |  |  | 3 | 5 |
| D | 95 | 34 | 8,5 | 7 | 5,6 | 11 | 38 | 15 | 2 | 40 | 15 | 2 |  |  | 1 | 2 |  |  |  |  | 4 | 6 | 2 | 3 |
| D | 111 | 50 | 11,9 | 7,95 | 6,6 | 15,4 | 51 | 16 | 2 | 52 | 16 | 2 |  |  | 2 |  |  |  |  |  | 5 |  | 3 | 4 |
| D | 113 | 50 | 10,6 | 7,7 | 6,7 | 14,8 | 50 | 16 | 2 | 51 | 19 | 2 |  |  | 3 |  |  |  |  |  | 6 |  | 3 | 4 |
| S | 41 | 38 | 10,2 | 7,7 | 5,85 | 15,1 | 48 | 15 | 1 | 50 | 15 | 1 | 1 | 2 |  |  | 2 | 3 | 4 | 6 |  |  | 2 | 3 |
| D | 219 | 38 | 9,95 | 7,65 | 6,05 | 13,1 | 45 | 15 | 1 | 48 | 17 | 1 |  |  | 1 | 3 |  |  |  |  | 4 | 6 | 2 | 2 |

* D: Dentergem, S: Semmerzake

**Supplementary Table S2.** Basic measurements and observations for the plants in the family plantation of Munte. Abbreviations of traits are in Tables 2 and 3.

| plantID | motherID | SL (mm) | SW (mm) | ST (mm) | FWW (mm) | LLs (mm) | LWs (mm) | LPs | LLl (mm) | LWl (mm) | LPl | BB 26ma | FO 26ma | BB 9ap | FO9 ap | BB 16ap | FO 16ap | FA 23se | FA 14oc |
| --- | --- | --- | --- | --- | --- | --- | --- | --- | --- | --- | --- | --- | --- | --- | --- | --- | --- | --- | --- |
| 156 | 1 | 8,95 | 7,4 | 5,85 | 13 | 43 | 19 | 1 | 43 | 28 | 3 | 2 | 2 | 3 | 3 | 5 | 6 | 1 | 3 |
| 169 | 1 | 10,6 | 8,75 | 6,3 | 15 | 48 | 20 | 1 | 64 | 37 | 3 | 1 | 2 | 2 | 2 | 3 | 6 | 1 | 2 |
| 173 | 1 | 8,55 | 7,2 | 5,6 | 13 | 39 | 18 | 1 | 40 | 23 | 3 | 1 | 3 | 3 | 3 | 4 | 6 | 1 | 2 |
| 131 | 1 | 11,5 | 8,25 | 6,45 | 17 | 47 | 22 | 1 | 51 | 26 | 2 | 1 | 2 | 2 | 2 | 4 | 6 | 1 | 2 |
| 176 | 1 | 8,3 | 7,15 | 5,2 | 12 | 45 | 19 | 1 | 49 | 28 | 2 | 2 | 1 | 3 | 2 | 5 | 5 | 1 | 2 |
| 159 | 1 | 10,4 | 7,8 | 5,4 | 13,5 | 35 | 19 | 1 | 51 | 34 | 4 | 1 | 2 | 3 | 2 | 4 | 6 | 2 | 3 |
| 162 | 2 | 9,05 | 7,45 | 5,8 | 14 | 40 | 18 | 1 | 59 | 45 | 3 | 1 | 1 | 2 | 2 | 3 | 6 | 2 | 3 |
| 172 | 2 | 9,15 | 6,6 | 4,85 | 13,5 | 37 | 17 | 1 | 56 | 38 | 4 | 1 | 1 | 2 | 2 | 4 | 6 | 2 | 3 |
| 132 | 2 | 10,1 | 7,8 | 5,6 | 14 | 37 | 16 | 1 | 50 | 19 | 1 | 1 | 2 | 3 | 3 | 4 | 6 | 1 | 2 |
| 142 | 2 | 10,6 | 7,5 | 5,7 | 14 | 48 | 16 | 1 | 60 | 32 | 1 | 2 | 2 | 3 | 2 | 4 | 6 | 2 | 3 |
| 148 | 2 | 10,45 | 7,85 | 5,85 | 14,5 | 52 | 20 | 1 | 60 | 28 | 1 | 3 | 2 | 4 | 2 | 5 | 6 | 1 | 2 |
| 150 | 2 | 8,95 | 7,2 | 5,65 | 14,5 | 37 | 19 | 2 | 43 | 29 | 4 | 1 | 2 | 2 | 2 | 4 | 5 | 1 | 3 |
| 180 | 2 | 10,55 | 7,7 | 5,55 | 13,5 | 44 | 19 | 1 | 50 | 21 | 2 | 1 | 1 | 2 | 2 | 4 | 6 | 2 | 2 |
| 170 | 2 | 8,75 | 6,45 | 4,8 | 12 | 38 | 17 | 2 | 50 | 32 | 3 | 2 | 2 | 3 | 2 | 5 | 6 | 2 | 3 |
| 121 | 3 | 10,1 | 7,2 | 5,5 | 15 | 53 | 22 | 1 | 45 | 29 | 4 | 2 | 2 | 4 | 3 | 6 | 6 | 1 | 2 |
| 149 | 3 | 8,05 | 6,55 | 5,05 | 12 | 30 | 13 | 2 | 32 | 20 | 3 | 1 | 2 | 2 | 2 | 4 | 4 | 3 | 3 |
| 128 | 4 | 9,1 | 7,3 | 5,65 | 14 | 37 | 16 | 1 | 57 | 40 | 3 | 1 | 2 | 2 | 2 | 4 | 5 | 1 | 2 |
| 129 | 4 | 9,5 | 7,8 | 6,25 | 14 | 32 | 13 | 1 | 45 | 27 | 4 | 2 | 2 | 3 | 2 | 4 | 6 | 1 | 2 |
| 160 | 4 | 8,7 | 7 | 5,05 | 12 | 35 | 14 | 1 | 38 | 20 | 4 | 1 | 1 | 2 | 2 | 4 | 6 | 2 | 4 |
| 161 | 4 | 10 | 7,3 | 5,45 | 13,5 | 38 | 16 | 1 | 41 | 27 | 2 | 1 | 2 | 2 | 2 | 4 | 6 | 1 | 2 |
| 134 | 4 | 11,2 | 8,6 | 6,35 | 15 | 33 | 18 | 1 | 70 | 38 | 4 | 1 | 2 | 2 | 2 | 4 | 6 | 1 | 2 |
| 140 | 4 | 9,6 | 8,5 | 6,5 | 14,5 | 48 | 20 | 1 | 48 | 33 | 3 | 2 | 1 | 3 | 2 | 4 | 5 | 1 | 3 |
| 164 | 4 | 8,25 | 7,75 | 5,85 | 13 | 34 | 11 | 1 | 47 | 22 | 4 | 1 | 2 | 1 | 2 | 4 | 6 | 2 | 3 |
| 122 | 5 | 11,1 | 9,3 | 6,65 | 17 | 47 | 20 | 1 | 60 | 33 | 4 | 1 | 2 | 2 | 2 | 4 | 6 | 1 | 3 |
| 138 | 5 | 10,8 | 9,2 | 6,8 | 18 | 42 | 21 | 2 | 48 | 40 | 4 | 1 | 2 | 3 | 2 | 5 | 6 | 2 | 3 |
| 163 | 5 |  |  |  | 18 | 44 | 25 | 2 | 70 | 46 | 3 | 1 | 2 | 3 | 2 | 5 | 5 | 1 | 2 |
| 153 | 6 | 17 | 10 | 7 | 23 | 63 | 27 | 2 | 60 | 30 | 3 | 1 | 2 | 3 | 2 | 4 | 6 | 1 | 2 |
| 130 | 6 | 10,15 | 9,4 | 7,8 | 19,5 | 39 | 19 | 1 | 40 | 23 | 5 | 2 | 3 | 4 | 4 | 5 | 6 | 1 | 2 |
| 126 | 6 | 11,41 | 9,2 | 6,85 | 17,5 | 41 | 22 | 2 | 60 | 35 | 2 | 1 | 3 | 3 | 2 | 5 | 5 | 2 | 4 |
| 178 | 6 |  |  |  |  | 58 | 26 | 4 | 56 | 31 | 5 | 1 | 1 | 3 | 2 | 6 | 6 | 2 | 3 |
| 167 | 6 | 20,5 | 12 | 6,9 | 32,5 | 45 | 28 | 2 | 58 | 43 | 3 | 1 | 2 | 4 | 2 | 6 | 6 | 3 | 3 |
| 165 | 7 | 13,55 | 9,3 | 6,8 | 20 | 45 | 13 | 1 | 56 | 39 | 3 | 1 | 2 | 2 | 2 | 4 | 6 | 2 | 3 |
| 136 | 7 | 14,8 | 11,2 | 7,06 | 20 | 46 | 21 | 1 | 56 | 36 | 3 | 2 | 3 | 3 | 3 | 5 | 6 | 2 | 4 |
| 139 | 7 | 9,7 | 8,2 | 5,9 | 16,5 | 45 | 20 | 1 | 55 | 32 | 4 | 1 | 2 | 3 | 3 | 4 | 6 | 2 | 4 |
| 141 | 7 |  |  |  |  | 48 | 22 | 3 | 70 | 48 | 4 | 2 | 1 | 4 |  | 5 |  | 2 | 4 |
| 143 | 7 | 14 | 8,5 | 6 | 20 |  |  |  |  |  |  | 2 | 2 | 2 | 2 | 4 | 6 | 2 | 4 |
| 144 | 7 | 11,5 | 10 | 5,75 | 20 | 35 | 16 | 1 | 42 | 38 | 5 | 1 | 1 | 3 | 2 | 4 | 6 | 2 | 4 |
| 175 | 7 | 16,85 | 13,8 | 9,95 | 22 | 50 | 30 | 2 | 85 | 58 | 3 | 2 | 3 | 4 | 3 | 6 | 6 | 3 | 4 |
| 177 | 7 | 12,9 | 10,1 | 7,05 | 23 | 45 | 26 | 2 | 62 | 35 | 2 | 1 | 3 | 3 | 4 | 5 | 6 | 2 | 3 |
| 133 | 8 | 10,25 | 8,35 | 6,55 | 14,5 | 40 | 17 | 1 | 64 | 42 | 3 | 2 | 2 | 4 | 3 | 5 | 6 | 1 | 2 |
| 145 | 8 | 7,85 | 6,9 | 5,55 | 13 | 39 | 15 | 1 | 55 | 32 | 3 | 1 | 1 | 3 | 2 | 4 | 6 | 2 | 3 |
| 123 | 8 | 10,1 | 8,45 | 6,3 | 13,5 | 36 | 14 | 1 | 52 | 25 | 3 | 1 | 2 | 2 | 2 | 4 | 6 | 2 | 3 |
| 124 | 8 | 8,2 | 6,65 | 5,05 | 17 | 53 | 32 | 2 | 42 | 24 | 4 | 2 | 2 | 3 | 3 | 5 | 6 | 2 | 3 |
| 125 | 8 | 10,05 | 7,9 | 5,65 | 13,5 | 40 | 17 | 1 | 56 | 25 | 1 | 1 | 2 | 3 | 2 | 4 | 6 | 2 | 3 |
| 127 | 8 | 12 | 9,5 | 7 | 19 | 53 | 22 | 3 | 65 | 32 | 4 | 1 | 3 | 3 | 3 | 5 | 6 | 2 | 3 |
| 171 | 8 | 9,25 | 7,65 | 5,75 | 13 | 41 | 18 | 1 | 42 | 27 | 3 | 2 | 2 | 3 | 3 | 4 | 6 | 2 | 3 |
